# In Vitro Assessment of the Impact of Industrial Processes on the Gastrointestinal Digestion of Milk Protein Matrices Using the INFOGEST Protocol

**DOI:** 10.3390/foods9111580

**Published:** 2020-10-30

**Authors:** Nathalie Atallah, Barbara Deracinois, Audrey Boulier, Alain Baniel, Delphine Jouan-Rimbaud Bouveresse, Rozenn Ravallec, Christophe Flahaut, Benoit Cudennec

**Affiliations:** 1 UMR Transfrontalière BioEcoAgro N° 1158, University Lille, INRAE, University Liège, UPJV, YNCREA, University Artois, University Littoral Côte d’Opale, ICV—Institut Charles Viollette, F-59000 Lille, France; nathalie.atallah@agroparistech.fr (N.A.); barbara.deracinois@univ-lille.fr (B.D.); rozenn.ravallec@univ-lille.fr (R.R.); christophe.flahaut@univ-artois.fr (C.F.); 2 Ingredia S.A. 51 Av. Lobbedez—CS 60946, 62033 Arras CEDEX, France; a.boulier@ingredia.com (A.B.); a.baniel@ingredia.com (A.B.); 3 AgroParisTech, Université Paris-Saclay, INRAE, UMR PNCA, 75005 Paris, France; delphine.bouveresse@agroparistech.fr

**Keywords:** in vitro digestion, milk proteins, industrial processes, protocol optimization, peptidomics

## Abstract

The goal of this study was to determine the impact of industrial processes on the digestion of six milk protein matrices using the harmonized INFOGEST in vitro static digestion protocol. First, this method was optimized to simple protein matrices using sodium dodecyl sulfate-polyacrylamide gel electrophoresis (SDS-PAGE) and size exclusion chromatography (SEC) to compare the intestinal protein hydrolysis obtained with increasing quantities of pancreatin. Similar results were achieved with the originally required pancreatin amount (trypsin activity of 100 U.mL^−1^) and with a quantity of pancreatin equivalent to a trypsin activity of 27.3 U.mL^−1^, which was thus used to perform the in vitro digestion of the milk matrices. Molecular weight profiles, peptide heterogeneity from LC-MS/MS data, calcium, free amino acid, and peptide concentrations were determined in the gastric and intestinal phases to compare the milk protein digests. Results showed that the industrial process affected not only the protein distribution of the matrices but also most likely the protein structures. Indeed, differences arose in terms of peptide populations generated when the caseins were reticulated or when their calcium concentrations were reduced.

## 1. Introduction

Milk proteins have been widely used in the food industry for the past 50 years due to the wide array of functions that they exhibit as food ingredients. Indeed, milk proteins are known not only for their nutritional value but also for their physicochemical and physiological properties [1]. Bovine milk proteins can be differentiated into two categories: micellar caseins and native whey proteins. Caseins are flexible phosphorylated proteins that are distinguished into four classes known as α-_S1_ α-_S2_, β-, and κ-caseins, which interact together to form a micelle. The structure of the casein micelle has been a subject of debates for years, and while no consensus has been agreed upon, two models are currently widespread: the submicellar and the nanocluster models [2]. The submicellar hypothesis formulates that the proteins in sodium caseinates, which characterize the protein extracts after removal of the calcium phosphate, form small aggregates through noncovalent interactions. These small aggregates, called submicelles, are linked together through small domains of calcium phosphate to form the casein micelles [2]. The nanocluster model suggests that the phosphopeptides present in β-caseins bind to and stabilize the calcium phosphate present in solutions in order to form nanoclusters. These calcium phosphate nanoclusters then interact with the more highly phosphorylated caseins (α-_S1_ and α-_S2_) to form the micellar particles. Both models agree that the κ-caseins are found on the surface of the micelles in a hairy layer, providing stability and limiting the growth of the micelle [2]. Whey proteins are globular proteins and are constituted of β-lactoglobulin, α-lactalbumin, serum albumin, immunoglobulins, lactoferrin, and other minor fractions [3]. 

It has already been shown in human trials that micellar caseins have a slow gastric emptying rate and are digested in up to six hours, whereas whey proteins have a fast gastric emptying rate and are digested in two hours [4,5]. This difference is explained by physicochemical properties specific to micellar casein and whey proteins. Indeed, caseins can form curds in the stomach, which delay gastric emptying, whereas whey proteins remain soluble in acidic conditions and are emptied rapidly from the stomach [5]. However, it is important to note that differences in digestion cannot only be due to the nature of a protein. Indeed, a given protein can be digested differently if it has been subjected to modifications during industrial processing [6].

Processes such as heating, for example, are known to denature whey proteins, making them more digestible [6]. In the case of caseins, acidification processes are known to affect their conformation, turning them into what are known as sodium caseinates, whereas other processes such as membrane filtration are known to keep them intact. The structural differences between the two processes can be seen by scanning electron microscopy (SEM), where sodium caseinates are shown to have irregular shapes and a porous structure [7]. On the contrary, SEM shows that, when processes such as membrane filtration are applied, the integrity of the casein micelle is preserved [8]. 

To date, there is little evidence linking a protein conformation to the way that is it digested. In a study conducted using a dynamic human gastric simulator, numerous milk matrices consisting of whey and caseins were tested [9]. The authors reported that milk protein concentrate and skim milk powder, which both contained casein micelles, formed curds early in digestion and at a pH > 6. However, calcium depleted milk protein concentrates, sodium caseinates formed curds 40 min into digestion at a pH < 5, and the structure of the curds were looser and lighter in weight. When they compared heated and nonheated whey protein isolates, they noticed that β-lactoglobulin was not hydrolyzed in the nonheated whey protein isolate, whereas it was hydrolyzed quickly in the heated isolate [9]. This study thus shows the impact of a protein’s structure, notably that of micellar caseins, and the influence of its processing on the way that it is digested.

As static digestion methods are less costly, less labor-intensive, faster than dynamic ones, and more ethical friendly than in vivo studies, it is often the method of choice for screening purposes. Moreover, since a harmonized protocol was developed by Minekus et al. in 2014 to improve the comparability of results between laboratories [10], static digestion methods have been further validated when compared to in vivo trials [11,12,13]. In this context of protein digestion understanding, this study aimed to compare the gastric and intestinal peptidomes of six milk matrices with varying ratios of protein categories and obtained with different industrial processes using the consensual INFOGEST static in vitro model simulating the gastrointestinal digestion and coming from the COST Action FA1005 INFOGEST (https://www.cost-infogest.eu/) [10]. The comparisons were made using size-exclusion chromatography (SEC) and mass spectrometry data (LC-MS/MS) among other analytical tools. However, in order to better compare our milk matrices, a preliminary aim of this work was to optimize the concentration of pancreatin in the INFOGEST protocol to adapt it to our simple milk matrices made up mostly of proteins.

## 2. Materials and Methods 

### 2.1. Materials

The milk matrices were provided in powder form by Ingredia, Arras, France. Porcine pepsin (EC 3.4.23.1, from porcine gastric mucosa, 250 units.mg^−1^ solid), pancreatin from porcine pancreas (EC number 232-468-9), and all other reagents were purchased from Sigma-Aldrich (Saint-Quentin Fallavier, France).

#### 2.1.1. Characteristics of the Milk Matrices

A total of 6 milk protein matrices (where C and W signify casein and whey, respectively) obtained by filtration and with different characteristics were digested in this study. Briefly, four of these matrices had the same concentration of caseins, three of which differed in calcium concentration levels (C1, C2, and C2_lowCa2+_) and one of which contained caseins that were reticulated by an enzymatic process (C3). One matrix (W) was constituted of whey proteins, and the other contained the same casein-to-whey ratio as that present in bovine milk (CW). The differences and similarities between the matrices are expressed in % in more details in Table 1. All of the matrices were rehydrated in water at 10% total nitrogen content before digestion. All of the matrices were provided by Ingredia.

#### 2.1.2. Estimation of Free Calcium Concentrations

The milk matrices were rehydrated in 100 mL of water (100 g.L^−1^) and then filtered in a mineral cartridge filter (Macrosep 10 K, Pall Biotech, Saint-Germain-en-Laye, France). After filtration, the suspension was centrifuged at a temperature of −20 °C and at 5000× *g* for 90 min in order to collect at least 5 g of filtrate. Calcium concentration levels were then estimated by atomic absorption spectrophotometry. Briefly, a solution of lanthanum (25 g.L^−1^); HCl (1% in H_2_O); and standard solutions of calcium, sodium, potassium, and magnesium; and the milk matrices were injected into a dual sample introduction pump system (SIPS 20, Agilent Technologies, Les Ulis, France). After setting up the spectrophotometer (VARIAN AA 240FS, Agilent Technologies), the emission intensity was measured for all the standards and samples with the software spectra 5.1 PRO. The calcium concentrations in the samples were determined according to a calibration curve reflecting absorbance vs. calcium, magnesium, and potassium concentrations. Calcium concentration levels were then evaluated in the oral, gastric, and intestinal phases according to dilution factors.

### 2.2. Static In Vitro Gastrointestinal Digestion of the Milk Matrices

The milk matrices were digested according to the INFOGEST harmonized static digestion protocol setup by Minekus et al. [10] but with some modifications in the concentration of pancreatin to better adapt it to the studied matrices (refer to Section 3.1.). Briefly, the milk matrices were digested in a single reactor at 37 °C under agitation. The first three steps of the adult gastrointestinal digestion were simulated: the oral phase, the gastric phase, and the intestinal phase. After rehydration in water at 10% total nitrogen content for all matrices, 8 mL of the matrix (or 8 mL of water for blank digestions) was mixed with an equivalent volume of simulated salivary fluid (SSF) for 2 min without amylase. Then, the mixture was diluted by half by the addition of a solution of simulated gastric fluid (SGF) and pepsin was added (2000 U/mL^−1^). The mixture was incubated for 120 min. Digestion samples of 2 mL were collected at different time points (30, 60, 90, and 120 min). The pH of the collected samples was raised to 7, and the samples were then heated at 90 °C for 10 min. After 2 h of gastric digestion, the pH was adjusted to 7 and the intestinal phase was carried out by adding the same volume (20 mL) of simulated intestinal fluid (SIF) to the gastric phase. Porcine pancreatin (trypsin activity of 27.3 U/mL^−1^) was added at the beginning of the intestinal phase. Pepsin and trypsin activities were measured using the methods described in the INFOGEST protocol. No bile was added due to the absence of lipids in the matrices. As in the gastric phase, samples of intestinal digests were collected at different time points (30, 60, 90, and 120 min) and heated at 90 °C for 10 min. Each matrix was digested 3 times to obtain biological replicates. The samples were all diluted to obtain a uniform concentration of 9.375 mg/mL^−1^ in the gastric and intestinal phases for analytical comparisons.

### 2.3. Biochemical Characterization

#### 2.3.1. Peptide Concentration Determination

The peptides present in the sample were quantified using the Folin-Ciocalteu (FC) reagent (ref F9252, Sigma-Aldrich). Briefly, 200 µL of extracted peptides were added to 500 µL of sodium carbonate solution (500 mM) and to 100 µL of FC reagent. After incubation of the samples at a temperature of 37 °C for 30 min in the dark, the peptide amount was determined by measuring the optical density at 750 nm (OD_750_). A peptide digest standard (Peptide digest assay standard, ThermoFisher Scientific, Waltham, USA) was used to set up the standard curve.

#### 2.3.2. Quantification of Free Amino Acids

The free amino acids were quantified for all replicates at the end of the gastric and intestinal phases. After precipitation of the samples in sulfosalicylic acid, the pH was decreased to 2.2. The amino acids were then separated by ion exchange chromatography and assayed using the ninhydrin method published by Bidlingmeyer et al. [14]. The detection of free amino acids was performed at 570 nm.

### 2.4. Peptide Molecular Weight Profiles

The molecular weight profiles of the matrices were obtained by size exclusion chromatography (SEC) based on fast protein liquid chromatography (FPLC) using a Superdex Peptide 10/300 GL column (10 × 300 − 310 mm, 13 µm, GE Healthcare, Little Chalfont, UK) coupled to an AKTA Purifier Protein Purification System (GE Healthcare). Twenty-five µL of the sample were injected under isocratic elution conditions with a 30% acetonitrile and 0.1% trifluoroacetic acid (TFA) solvent at a flow rate of 0.5 mL.min^−1^ for 60 min. Absorbance was monitored at 214 nm. The column was calibrated using internal standard peptides (cytochrome C, 12,400 Da; aprotinin, 6500 Da; vitamin B12, 1355 Da; and glutathione 307.3 Da) and albumin to determine the column dead volume. The peptide molecular weight distribution was obtained using the relationship between Log of molecular weight of standard peptides and the elution volume. Six molecular weight (Da) ranges were determined, two corresponding to high molecular weight peptides/proteins (30,000 < molecular weight (MW) < 10,000 and 10,000 < MW < 4000), two corresponding to intermediary molecular weight peptides (4000 < MW < 2000 and 2000 < MW < 700), and two corresponding to low molecular weight peptides (700 < MW < 300 and 300 < MW < 30). Peptide content estimation was then expressed as a percentage of the total profile area and referred to as area under the curve (AUC).

### 2.5. Gel Electrophoresis

Protein hydrolysis with different quantities of pancreatin was analyzed in the intestinal phase by SDS-PAGE following the Laemlli method [15]. Briefly, a 12.5% polyacrylamide resolving gel and a 4% stacking gel were used. The phase samples were diluted (1:2) in Laemmli buffer containing β-mercaptoethanol and sodium dodecyl sulfate (SDS) and then heated at 95 °C for 10 min. Fifteen µL of the samples as well as 10 µL of a solution of molecular weight markers (Bio-Rad, Marnes-la-Coquette, France) were deposited on the gel. The migration of peptides and proteins was performed for one hour in a base buffer containing Tris (30.3 g.L^−1^), glycine (144 g.L^−1^), and SDS (10 g.L^−1^), pH 8.3. A voltage of 100 V was applied during the first 10 min and then raised to 200 V until the end of migration. Thereafter, the gels were stained with colloidal Coomassie Blue.

### 2.6. Peptide Identification by HPLC-ESI-qTOF-MS/MS and Database Search

Digestion samples collected at the end of the gastric and intestinal phases were first purified in order to remove remaining salts and sugars by adding 2 mL of the samples to a C_18_ solid phase extraction (SPE) with Bond Elut C_18_ 1000 mg minicolumns (Agilent Technologies). The C_18_-retained peptides were then eluted with 3 mL of 80% (*v/v*) acetonitrile (ACN), 20% (*v/v*) water, and 0.1% (*v/v*) TFA. The C18-retained peptide fractions were dried by centrifugal evaporation (miVac Centrifugal Vacuum Concentrators, Gene Vac, Ipswich, UK) for 3 h at 40 °C and stored in the dark at room temperature until further analysis.

The dried peptides were dissolved in 100 µL H_2_O and 0.1% TFA, submitted to 3 vortex/sonication cycles (20 s/20 s), and centrifuged for 10 min at 9300 × *g*. Ten µL of supernatants were collected for separation and identification of peptides by peptidomics approach. Two distinct quality control (QC) samples corresponding to equivolume mixtures of the matrix triplicates at the end of the gastric and intestinal phases were also injected at the beginning, the middle, and the end of the HPLC-MS/MS analysis session (one HPLC-MS/MS session for 18 gastric samples + 3 gastric QCs and one for the 18 intestinal samples + 3 intestinal QCs). All HPLC-MS/MS analyses were performed in triplicate. Peptides of supernatants and QCs were separated by chromatography on an ACQUITY HPLC system (Waters, Manchester, UK) using a C18 Kinetex column (150 × 4.6 mm, 2.6 µm, 100 Å, Phenomenex, Le Pecq, France) by running a discontinuous linear gradient of ACN containing 0.1% formic acid (FA) (5–15% ACN over 30 min, 15–30% ACN over 60 min, 30–50% ACN over 10 min, and 50–95% ACN over 10 min) at a flow rate of 500 µL.min^−1^. The HPLC eluent was then directly electrosprayed at the end of the column at a voltage of 3 kV, using a desolvation gas (N_2_) at a flow of 600 L.h^−1^, a nebulizer gas flow of 2.5 bar, and a temperature of 300 °C. The HPLC was coupled to a SYNAPT-G2-Si mass spectrometer (Waters) previously calibrated using a sodium formate solution. Mass spectrometry measurements were made in sensitivity- and positive-mode using the proprietary MassLynx software (version 4.1, Waters). MS and MS/MS analyses were performed in a data-dependent analysis (DDA) mode, and mass data were collected in the measurement range of 50 to 1700 *m/z* using lock mass correction with 556.632 *m/z*, corresponding to simply charged leucine enkephalin. A maximum of 10 precursor ions with an intensity threshold of 10,000 counts was selected to be fragmented by collision-induced dissociation (CID) at an energy collision of 8 V to 9 V for low molecular weight ions and at a range of 40 V to 90 V for high molecular weight ions.

Database searches were performed on PEAKS studio v8.5 (Bioinformatics Solutions Inc., Waterloo, Canada) using the UniProt database restricted to the *Bos taurus* organism (downloaded on the 10th of September, 2018—6002 entries). A mass tolerance of 35 ppm, 3 missing cleavage sites, no specific enzyme, and an MS/MS tolerance of 0.2 Da were allowed. Variable methionine oxidation was also considered. The relevance of peptide identities was considered according to the identification generated by PEAKS studio 8.5 (*p <* 0.05) and a false discovery rate (FDR) strictly inferior to 1%. No minimum length of amino acid sequence for peptide identification was set, and the peptides identified by de novo approach were not considered.

### 2.7. Bioinformatics Retreatment of Chromatographic and Mass Spectrometry Data

Raw data of all HPLC-MS/MS runs were imported to Progenesis QI for proteomics 4.1 software (Nonlinear Dynamics, Newcastle upon Tyne, UK) using the following parameters: (i) data alignment was automatically managed by Progenesis software using one of all QC runs as reference; (ii) a slight manual alignment was performed to optimize the HPLC-MS/MS run alignment; and (iii) all runs were used for peak picking with a peak picking low limit of 5000, a maximum charge of +5, and retention times defined between 5 to 50 min (iv) when chromatographic peak width was not limited and (v) when data normalization was automatically performed for the statistical analysis in main components (principal component analysis (PCA)). Relative quantification of proteins was based on the 3 most abundant N-peptides of a given protein. Mass data were exported from Progenesis 4.1 to PEAKS Studio 8.5 for peptide identifications (performed as aforementioned), and these latter were reimported to Progenesis 4.1. The criteria selection for statistical comparison of mass signals of HPLC-MS/MS runs were set as follow: (i) intensity threshold = minimum of 1 × 10^5^ and (ii) an ANOVA *p*-value < 0.005.

### 2.8. Amino Acid Occurrence

In order to envision peptide abundance, the data were exported from PEAKS Studio 8.5 to a home-built Microsoft Excel heat map giving the amino acid occurrences in each major protein at the end of the gastric and intestinal phases. These were then exported to GraphPad Prism 8.2.1. The results were generated for the most abundant proteins in the casein and whey fractions of milk: α-S1 casein and β-lactoglobulin [16].

### 2.9. Multi Block-Based Statistical Analysis

Grouped data analyses were performed with in-house routines and the SAISIR© Toolbox [17] using MatLab 7.6.0 (The MathWorks, Natick, MA, USA). As the chemical and mass spectrometry data were sorted into tables with a different number of variables, a multi-block analysis known as common components and specific weight analysis (CCSWA) developed by Qannari et al. was implemented [18]. This method calculates a space that is common to all the tables referred to as common components (CC) space. After normalization of the tables, an iterative process is used to estimate the contribution (called salience) of each table in the construction of each common component. Significant differences in the values of saliences reflect that tables did not contribute equally to the construction of the common component in question.

## 3. Results

### 3.1. Optimization of the INFOGEST Digestion Protocol for Dairy Protein Matrices

**Figure 1:**
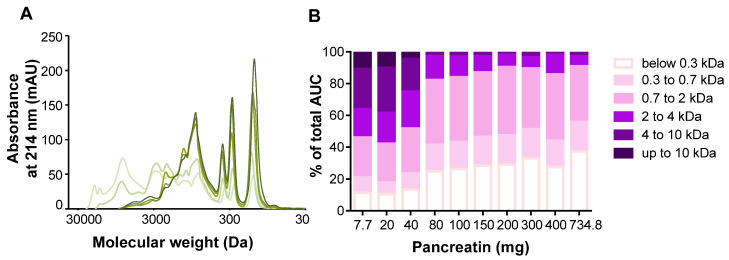
Molecular weight profiles and distributions of W intestinal digests: optimization of the static gastrointestinal digestion (SGID). (**A**) Peptide profiles were obtained by size exclusion chromatography-fast protein liquid chromatography (SEC-FPLC): 7.7, 20, 200, 400, and 734.8 mg of pancreatin (light to dark green curves). (**B**) The apparent molecular weight distribution of peptides, expressed in percentage of total area under the curve (AUC), was calculated from the linear regression relationship, which correlates the Log of known molecular weight standard peptides and the elution volume.

**Figure 2:**
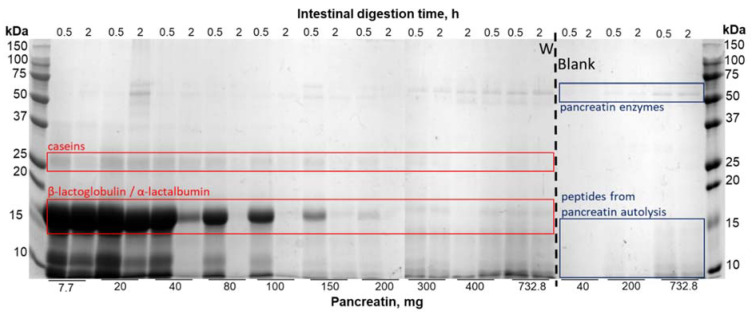
SDS-PAGE profiles of the native whey matrix (W) and of blank digestion (Blank) intestinal digests at the beginning (0.5 h) and end of the intestinal phase (2 h).

The optimization of the static in vitro digestion for dairy protein was performed with the whey protein isolate (W matrix) as whey proteins are more resistant to gastrointestinal enzymes [18]. Peptide molecular weight profiles and distributions of intestinal samples of the W matrix digested with increasing quantities of pancreatin are presented in Figure 1. According to the INFOGEST protocol, a concentration of 100 U.mL^−1^ of pancreatin was required (based on trypsin activity) to digest the matrices. This would have been equal to 732.8 mg of pancreatin (or 18.32 mg.mL^−1^ of digest), giving us an enzyme/substrate (*w/w*) ratio close to 1 (732.8 mg of pancreatin for 800 mg of protein). The molecular weight profiles showed that, for quantities of 7.7 mg and 20 mg of pancreatin, the shape of the profile differed greatly compared to quantities of 200 mg and above. Indeed, when smaller quantities of pancreatin were used, the extent of the curve was wider as a great proportion of intermediary and high molecular weight peptides was generated, whereas a majority of smaller peptides between 30 and 3000 Da was generated for quantities of 200 mg and above (Figure 1A). In accordance with the molecular weight profiles, quantities of 7.7 mg and 20 mg of pancreatin generated half of the peptide population comprised between 2 kDa and 10 kDa, whereas the other half was made up of low molecular weights between 2 and 0.3 kDa. With the addition of 40 mg of pancreatin, the proportions were similar to those of 7.7 and 20 mg of pancreatin but with a slightly higher proportion of lower molecular weights. From 80 to 200 mg of pancreatin, the proportion of molecular weights were comparable, as molecular weights between 0.3 and 2 kDa made up the majority of the peptide population. Starting from 300 mg of pancreatin and despite the majority of the peptide population being constituted of low molecular weights, an increase in the proportion of molecular weights between 2 and 4 kDa was observed. This increase was presumably due to the contribution of pancreatin peptides generated by autolysis (Figure 1B). These results are in accordance with the SDS-PAGE profiles obtained during intestinal digestion of the W matrix and intestinal blank digestion at different concentrations of pancreatin. A band most likely representing β-lactoglobulin was present at the end of the intestinal phase when small quantities of pancreatin were used (Figure 2). This band was still present at the end of 2-h digestion when quantities of 7.7, 20, and 40 mg were used. However, when a quantity of 80 mg of pancreatin was used, this protein band disappeared at the end of the intestinal phase and only completely disappear after 120 min of the intestinal digestion when a quantity of 200 mg of pancreatin was used. When higher amounts going from 300 mg to 732.8 mg were tested, the same result was observed: the band disappearing 120 min into intestinal digestion. Moreover, a band at 25 kDa, presumably representing residual β-casein, was still present at the end of the intestinal phase when quantities inferior to 200 mg of pancreatin were added. The assumption that this band representedβ-casein not only was based on the protein size but also was later confirmed with the protein identification results obtained by LC-MS/MS (Supplementary Table S1). It can thus be argued that, despite the extraction process used to obtain the W matrix, residues of casein were still present. Thus, the residual casein bands started to disappear at the end of the intestinal phase when amounts equal or superior to 200 mg were added. The blank digestions further prove that the identified protein at 15 kDa is β-lactoglobulin as the band corresponding to its size is no longer present in the absence of the W matrix. Finally, the blank digestions lead to the conclusion that the bands observed around 50 kDa correspond to enzymes present in pancreatin. Moreover, the intensity of the bands inferior to 15 kDa increases starting from 400 mg of pancreatin, which can be attributed to its autolysis. Taken all together, the results described above and discussed later led us to perform the digestion of the dairy protein matrices with 200 mg of pancreatin, which equals to a trypsin activity of 23.7 U.mL^−1^ of intestinal digest.

### 3.2. Comparison of the Gastric and Intestinal Digests of the Dairy Matrices with Size Exclusion Chromatography

**Figure 3:**
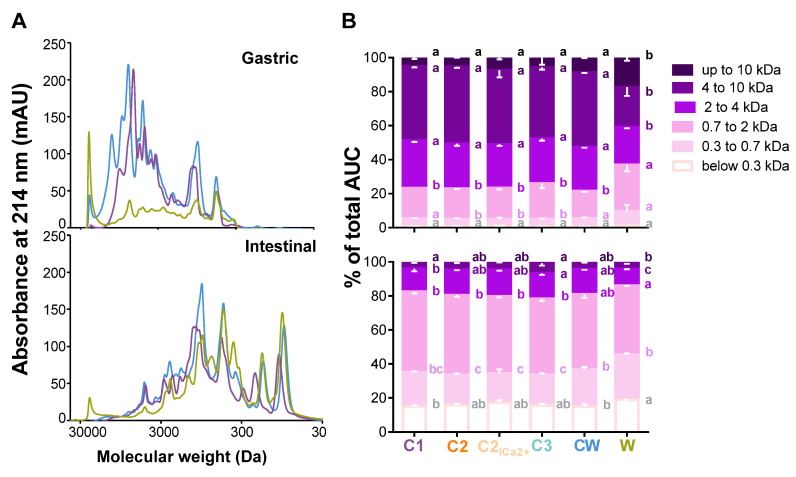
Molecular weight profiles and distributions of the gastric and intestinal digests of the different matrices: (**A**) peptide profiles were obtained by size-exclusion chromatography-fast protein liquid chromatography (SEC-FPLC): W (green curve), CW (dark blue curve), C1 (purple curve), 2-h gastric digests (top), and 2-h intestinal digests (bottom). (**B**) The mean (±SD) molecular weight distribution of peptides in the different compartment of the SGID, expressed in percentage of total area under the curve (AUC), was calculated from the linear regression relationship which correlates the Log of known molecular weight standard peptides and the elution volume. In a same molecular weight range, bars with different lowercase letters are significantly different (*p <* 0.05, one-way ANOVA).

Figure 3 presents the results obtained by the size-exclusion chromatography of the different final gastric and intestinal digests. The molecular profiles clearly show the impact of the composition of the matrices on hydrolysis. Indeed, in the gastric phase, the curve shape varied significantly between C1, CW, and W. The shape of W was flatter than that of CW and C1 and exhibited mainly low peaks, the highest one being for high molecular weights. C1 generated lower molecular weights than W, and CW exhibited the highest peaks and stretched from high to low molecular weights. At the end of the intestinal phase, some differences between all matrices persisted, but all the curves displayed a shift towards lower molecular weights due to hydrolysis (Figure 3A). The molecular profiles of all gastric and intestinal digests are presented in Figure S1 (Supplementary Figure S1). At the end of the gastric phase, the molecular weight distribution for all size categories was shown to be very similar for the matrices C2, C2_low Ca2+_, and C3 which are all concentrated in caseins. Indeed, they all exhibited a majority of peptides comprised between 4 to 10 kDa. C1 and CW differed from the other casein matrices as they had a significantly higher percentage of molecular weights comprised between 0.3 to 0.7 kDa. W stood out from the other matrices since the distribution of its molecular weights was more homogeneous in terms of size categories. Indeed, it had a significantly higher proportion of peptides weighing above 10 kDa and a significantly lower proportion of intermediate molecular weights. By the end of the intestinal phase, the repartition of molecular weights was still very similar for C2, C2_low Ca2+_, and C3 as they were significantly different in most size categories compared to C1, CW, and W. W was very different particularly for the size categories below 0.3 kDa, 0.3 to 0.7 kDa, and 0.7 to 2 kDa which represented a higher percentage of the AUC compared to all the casein matrices (C1, C2, C2_low Ca2+_, and C3). As for CW, the distribution of the molecular weights was again intermediary between that of the casein-enriched matrices and the W matrix. (Figure 3B).

### 3.3. HPLC-MS/MS Analysis of the Gastric and Intestinal Peptidomes of the Dairy Matrices

Triplicates of concentrated and desalted peptide samples of each 2-h gastric and 2-h intestinal digest and the associated QCs were subjected to an HPLC-MS/MS analysis (see the Materials and Methods section). The average numbers of MS scans and MS/MS scans were 4272 ± 684 and 12,479 ± 1216 for the 2-h gastric digestion HPLC-MS/MS runs, respectively, while the average number of MS scans and MS/MS scans were 3638 ± 1121 and 13,713 ± 2253 for the gastric–QC HPLC-MS/MS runs, respectively. In the same manner, the average number of MS scans and MS/MS scans were 5821 ± 491 and 9831 ± 872 for the 2-h intestinal digestion HPLC-MS/MS runs, respectively, while the average number of MS scans and MS/MS scans were 5964 ± 953 and 9567 ± 1686 for the intestinal–QC HPLC-MS/MS runs, respectively. The statistical values related to MS and MS/MS scan numbers demonstrated that no significant differences existed among the mass spectrometry data collected for all the matrix replicates after the gastric phase (*p =* 0.53, one-way ANOVA) and after the intestinal phase (*p =* 0.82, one-way ANOVA). HPLC-MS/MS raw data were imported in PEAKS Studio 8.5 software for peptide identifications using *Bos taurus*-restricted UniProt database. From each triplicate of HPLC-MS/MS runs, around 815 ± 109 peptides were identified (FDR < 1%) from all 2-h gastric digests. The average number of identified peptides from the triplicate of gastric–QC HPLC-MS/MS runs was 900 ± 140. Concomitantly, from each triplicate of HPLC-MS/MS runs, around 211 ± 46 peptides were identified (FDR < 1%) from all 2-h intestinal digests except for the C2 and C2 low Ca^2+^, where the number of identified peptides were close to 328 ± 68. The average number of identified peptides from the triplicate of intestinal–QC HPLC-MS/MS runs was 287 ± 3. It should be noted that the peptides composed of 2 to 5 amino acids were not considered due to the lack of certainty in their identifications.

**Figure 4:**
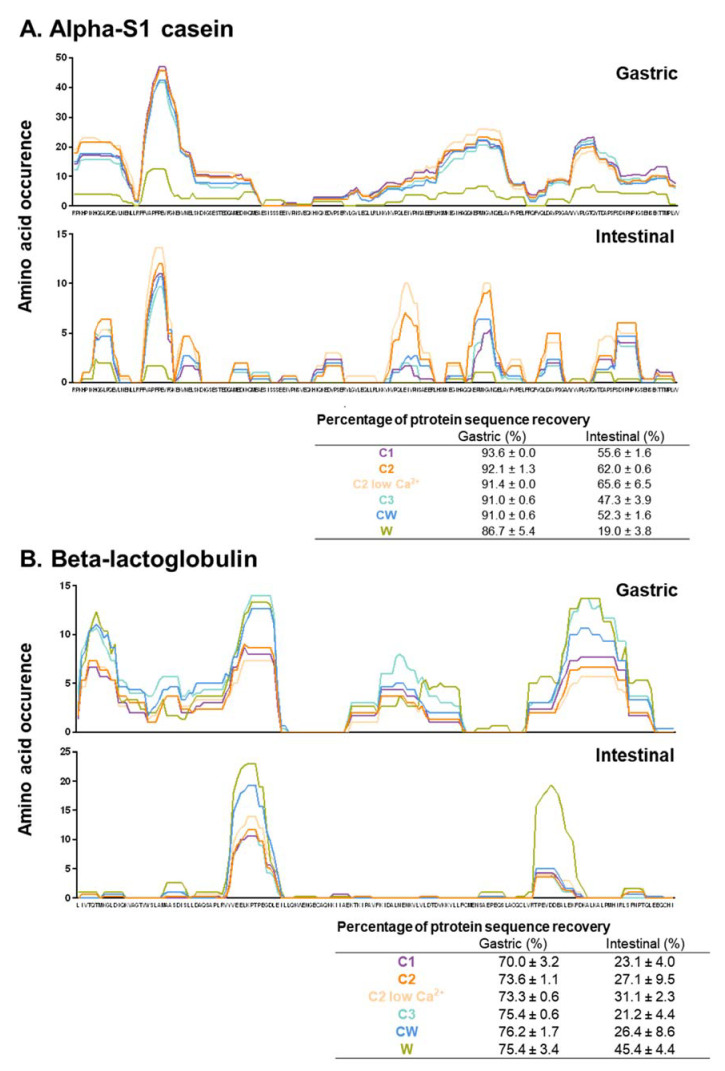
Peptide patterns of the different matrices at the end of the gastric and intestinal phases of the SGID: (**A**) alpha-S1 casein and (**B**) beta-lactoglobulin, with the x-axis representing the amino acid sequence of the different proteins and the y-axis representing amino acid occurrence.

From all identified peptides, the amino acid occurrences were calculated as described in the Materials and Methods section (Figure 4A,B), where the y-axis represents the amino acid occurrence and the x-axis represents the amino acid sequence of the protein. Figure 4A,B illustrates the amino acid occurrence obtained, following the different phases of digestion, for alpha-S1 casein and beta-lactoglobulin. Briefly, high amino acid occurrence at a given location on the protein backbone signifies that a substantial number of peptides of suitable size have been identified with certainty following the bioinformatics retreatment of HPLC-MS/MS raw data by PEAKS Studio 8.5. The substantial numbers of peptides identified at a same location of the protein backbone result in a high amino acid occurrence. In other words, high amino acid occurrence signifies that the concerned protein regions are resistant to enzyme action during the digestion phase. Whatever the digestion phase, the amino acid occurrence patterns of alpha-S1 casein (Figure 4A) were similar for all milk matrices with the same resistance regions but with different amino acid occurrences. Indeed, the matrix W is shown to have the lowest number of occurrences as it contains trace amounts of alpha S1 casein. When it comes to beta-lactoglobulin (Figure 4B), the same regions of sensitivity or resistance to the static gastrointestinal digestion (SGID) are again observed between all matrices, with the W matrix having the highest occurrence as it has the highest levels of beta-lactoglobulin.

HPLC-MS/MS raw data were also imported in Progenesis QI for proteomics (V4.1) software. HPLC-MS/MS run data were automatically and manually aligned, peak picking of mass signals was constrained (see the Materials and Methods section), data normalization was automatically performed, and the statistical comparison of mass signals was conducted using the filtering criteria described in the Materials and Methods section. Subsequently, PEAKS Studio-based peptide identities were reimported in Progenesis QI.

**Figure 5:**
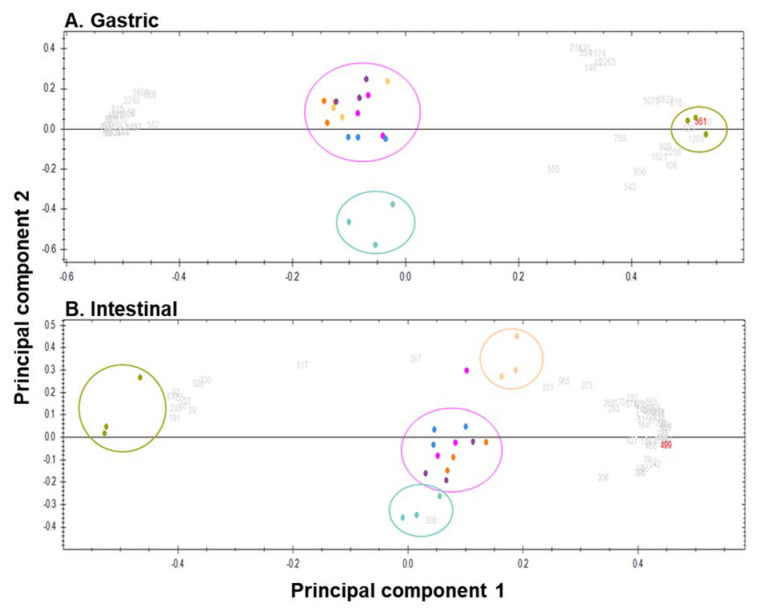
Principal component analysis of the most relevant identified peptides detected at the end of the gastric (**A**) and intestinal (**B**) phases of the SGID: grey and red numbers correspond to the identified peptide displaying a mass signal intensity > to 1 × 10^5^ and an ANOVA *p*-value < 0.005. Color circles are hand-marked to facilitate understanding and have no statistical significance. C1, purple; C2, dark orange; C2 low Ca2+, light orange; C3, light blue; CW, dark blue; W, green; and QC, pink.

Among the 2366 peak picked mass signals detected in all mingled dairy protein gastric digests which have an intensity threshold > 1 × 10^5^, 482 peptide ions displayed a quantitative difference with an ANOVA *p*-value inferior to 0.005, and finally, 47 identified peptide ions had both thresholds and displayed a positive false discovery rate (q-value) between 3.63 × 10^−11^ < q-value < 0.0033 and a power value between 0.964 and 0.9995 (Figure 5A). In the same manner, among the 13,919 peak picked mass signals detected in all mingled dairy protein intestinal digests, 579 peptide ions had an intensity threshold > 1 × 10^5^, 120 peptide ions displayed an ANOVA *p*-value inferior to 0.005, and finally, 63 identified peptide ions had both thresholds and displayed a positive false discovery rate (q-value) between 3.92 × 10^−12^ < q-value < 0.00142 and a power value between 0.950 and 0.9995 (Figure 5B). These latter values demonstrate that only the most instance and different peptide ions (#47 and #63) were selected for PCA. The first five dimensions of the PCA (Figure 5A,B) explain 93.49% and 95.40% of previously filtered mass signal variances of 2-h gastric and 2-h intestinal digests, respectively. Figure 5A displays the PCA generated using the first two dimensions (PC1 = 76.06% and PC2 = 10.05%) of filtered peptides identified at the end of the gastric phase where 3 distinct clusters appear. The more distant the groups, the more different they are in terms of peptide population. Color circles were hand-marked for clarity and have no statistical relevance. The central cluster gathered the 2-h gastric digests of all matrices (CW, C1, C2, and C2 low Ca^2+^) except for matrix C3, which formed the bottom cluster, and matrix W, on the right-hand side, which was largely distant from the two other clusters. All grey and red numbers depicted correspond to the identified peptides displaying a mass signal intensity > to 1 × 10^5^ and an ANOVA *p*-value < 0.005. Figure 5B represents the PCA generated using the first two dimensions (PC1 = 77.37% and PC2 = 10.17%) at the end of the intestinal phase where 4 clusters appear. A central cluster composed of matrices CW, C1, and C2 was close but distinguishable to the cluster formed by matrix C3 on PC2. The third cluster corresponded to the matrix C2 low Ca^2+^ (top right region of the PCA), while the fourth cluster corresponded to matrix W and, again, was largely distant from all other clusters.

Among the 63 identified peptides of interest in intestinal digests, 12 amino acid sequences were redundant. Table S1 gathers the 51 unique peptide sequences which are statistically distinguishable by their abundance among the 6 milk matrices. A unique peptide is defined as a peptide, irrespective of its length, that exists only in one protein of a proteome of interest despite the fact that this peptide may appear more than once in the same protein [19]. Table S1 is ordered according to (i) the matrix nature, (ii) the milk main proteins, (iii) the ANOVA *p*-value, and (iv) the maximum fold changed measured.

The C2 matrix contained, in highest mean normalized abundance, only 2 peptides from α-S1 casein and β-casein, which corresponded to the α-S1 phosphorylated peptide EIVPNpSAEE and a sodium adduct of the Cas-β peptide VYPFPGPIPN (not considered), while the C3 matrix contained, in highest mean normalized abundance, 4 peptides from the same proteins but without posttranslational modifications. The CW matrix contained 6 peptides, in highest mean normalized abundance, from α-lactalbumin, α-S1 casein, β-casein, and β-lactoglobulin (one of them was a sodium adduct peptide (β-casein: VVPPFLQPEV (E+21.98))). The W matrix contained 10 highest mean normalized abundance peptides mainly, as expected, from β-lactoglobulin and α-lactalbumin but 2 peptides from β-casein. Only one β-lactoglobulin peptide displayed a mass increment, suggesting that Gly is replaced by Val. Interestingly, the C2 low Ca^2+^ matrix contained, in highest mean normalized abundance, 29 peptides which came only from the 4 casein proteins (α-S1, α-S2, β-, and κ-casein). Among these 29 highest mean normalized abundance peptides, 4 were posttranslationally modified, 3 of which were serine-phosphorylated peptides (NMAINPpSKEN, KTVDMEpSTEV, and LNVPGEIVEpSL).

### 3.4. Statistical Comparisons of the Gastric and the Intestinal Digests of the Dairy Matrices

**Figure 6:**
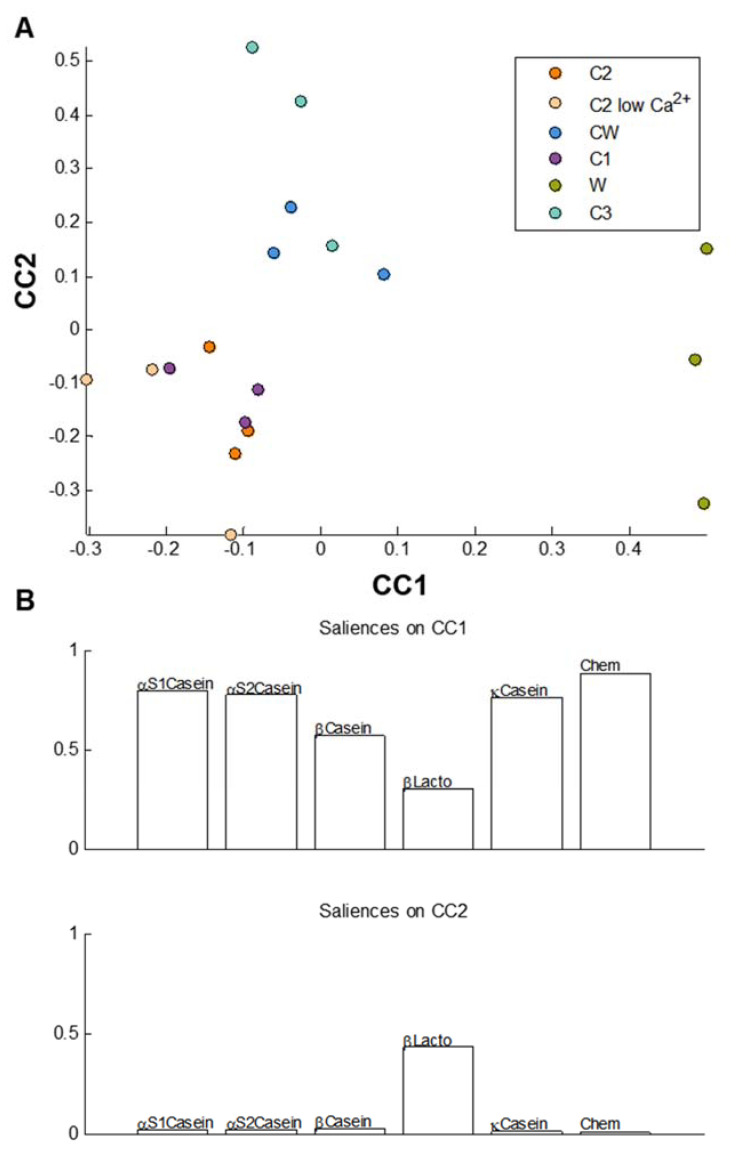
Common component and specific weight analysis (CCSWA) of the different matrices at the end of the gastric phase for all three replicates of the matrices: (**A**) CC1 vs. CC2 score plot and (**B**) saliences on CC1 and CC2.

To compare the milk matrices from every analytical standpoint, a multi-block analysis was applied (see the Materials and Methods section). Figure 6 represents the common components analysis between the matrices at the end of the gastric phase after incorporation of all the chemical data (AUC of SEC, peptide concentration, free amino acid concentration, and calcium concentrations) in one table (Table 2) and the amino acid occurrences for each main protein in respective tables.

The score plots in Figure 6A show that the W matrix can be distinguished from the others on the CC1 axis, as it is situated far away from the other matrices. This is linked to the results in Figure 4, where the W matrix is shown to have the lowest occurrences in amino acids obtained from α-_S1_ casein. The saliences (Figure 6B) show that CC1 was built based on information present in all the data tables, with the chemical data table contributing the most while β-lactoglobulin contributed the least. On the CC2 axis, most of the matrices seem to be grouped, except for the C3 and CW matrices, which are separated from the rest of the samples. The saliences show that CC2 was built exclusively based on information present in the β-lactoglobulin table.

**Figure 7:**
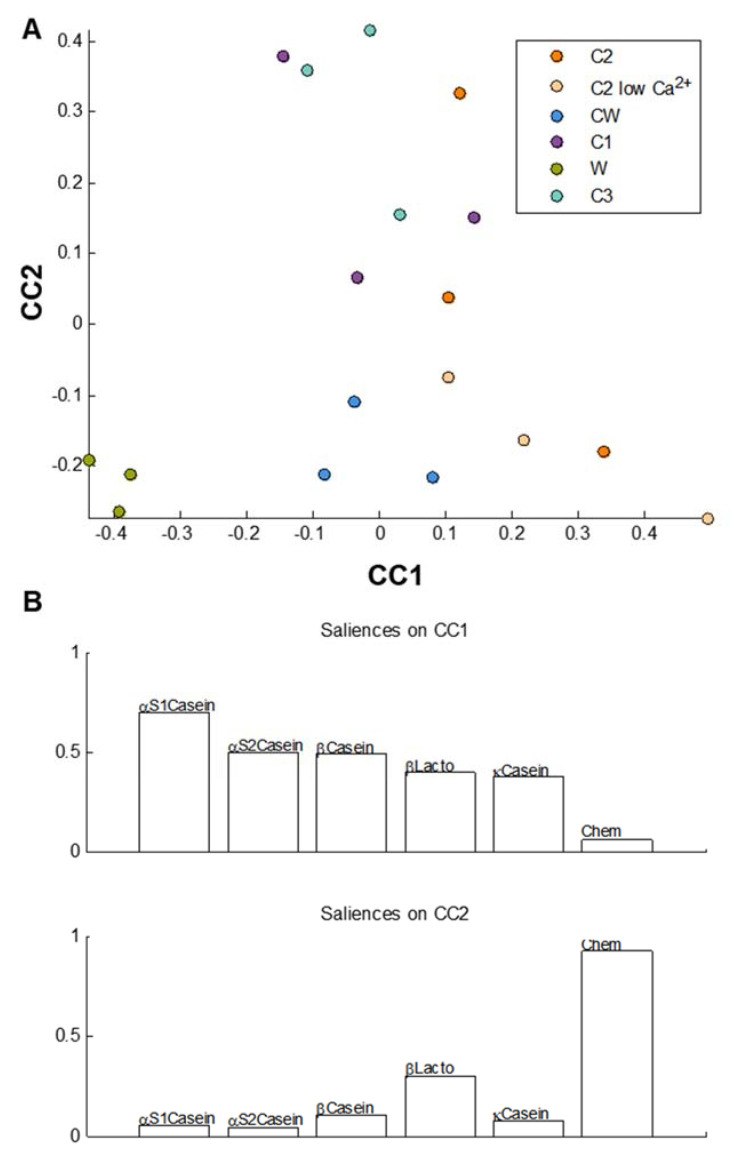
CCSWA of the different matrices at the end of the intestinal phase for all three replicates of the matrices: (**A**) CC1 vs. CC2 scores plot and (**B**) saliences on CC1 and CC2.

Figure 7 represents the common components analysis between the matrices at the end of the intestinal phase after incorporation of all the chemical data (AUC of SEC, peptide concentration, free amino acid concentration, and calcium concentrations) in Table 2 and the amino acid occurrences for each main protein in respective tables.

Figure 7A shows that the W matrix can be distinguished from the other matrices on the CC1 axis. The saliences show that information from each protein table was used to construct CC1; hence, it can be concluded that the W matrix was different from the other matrices because of variables present in all of the protein data tables. On the CC2 axis, the replicates are more scattered. Indeed, CC2 shows regrouping of the C1, C2, and C3 replicates on one hand and another regrouping of the CW, W, and C2 low Ca^2+^ replicates on the other hand. The salience graph (Figure 7B) shows that CC2 was built based on information present in the chemical data table.

## 4. Discussion

### 4.1. Optimization of the Harmonized Protocol

According to the protocol by Minekus et al. [10], a quantity of 732.8 mg of pancreatin (trypsin activity of 100 U.mL^−1^) was required to digest our liquid protein matrices. The protocol by Minekus et al. was developed as a general standardized practical static digestion method to improve comparability between different laboratories but can be modified to better suit certain research questions when necessary [10]. Since the protocol established by Minekus et al. targets a wide range of food matrices and since the matrices in our study were simple and only contained proteins, the quantity of pancreatin was optimized. To the best of our knowledge, no comparability study of the digestion profiles obtained with different concentrations of pancreatin was conducted. Despite being more cost effective, decreasing the quantity of pancreatin needed would allow for a clearer identification of peptidomes in mass spectrometry, which is a common analytical tool used for in vitro digestion studies. The matrix W was chosen to optimize the gastric in vitro digestion protocol because whey proteins are known to be resistant to gastric digestion, whereas caseins are sensitive to hydrolysis. Indeed, this is due to the globular tertiary structure of whey proteins, which makes them partly resistant to hydrolysis by pepsin, whereas caseins are easily hydrolyzed thanks to their flexible conformation [20] due to the low presence of secondary and tertiary structures. Taken collectively, the results obtained in the optimization protocol (Figure 1 and Figure 2) show that protein hydrolysis varies with increasing quantities of pancreatin until it reaches a point where it stabilizes at 200 mg (trypsin activity of 27.3 U.mL^−1^). Differences rise again, notably starting from 400 mg, and this seems to be due to pancreatin autolysis. Indeed, it has already been shown in previous studies [19,20] that autolysis of proteases present in pancreatin occurred and varied according to in vitro digestion conditions. This is especially true at high concentrations of pancreatin where the activity of certain proteases such as trypsin is lost more quickly than at lower concentrations in the presence of substrate [21]. This autolysis mechanism thus explains the increase in the proportion of peptides harboring apparent molecular weights comprised between 4 and 10 kDa (Figure 1) and the increase in band intensities for molecular weights inferior to 15 kDa (Figure 2) for quantities of pancreatin superior to 400 mg. Since the peptide profile (Figure 1A) of the W matrix was very similar to that obtained with the INFOGEST protocol’s initial concentration of 732.8 mg of pancreatin and as the SDS-PAGE profile showed complete hydrolysis of the matrix at the end of the intestinal phase, a quantity of 200 mg of pancreatin was chosen to conduct the rest of our experiments.

### 4.2. Comparisons of the Molecular Weights and Resistance Zones Between the Milk Matrices

All of the casein matrices were very similar in terms of peptide molecular weight profile and distribution of gastric and intestinal digests despite the different industrial processes applied to the matrices and the variability of calcium concentrations (Figure 3; data not shown for all casein matrices). Hydrolysis for all matrices (C1, C2, C2 low Ca^2+^, and C3) starts at the gastric phase, and the increase in lower molecular weights occurs at the end of the intestinal phase. Indeed, at this scale, the only visible differences are shown when the casein matrices were compared to the W and CW matrices. The W matrix behaved in a very distinctive way during gastric digestion due to its overall composition in whey proteins. As previously mentioned, whey proteins are resistant to gastric digestion, and our results go hand in hand with those of previous studies [11,12,13]. In these two studies, skim milk powder was digested according to the INFOGEST harmonized gastric digestion protocol. In both studies, the protein β-lactoglobulin was resistant to gastric digestion by pepsin and was still intact at the end of the gastric phase. The hydrolysis of α-lactalbumin and the caseins had however started during the gastric phase, and the generation of molecular weights of the W matrix at this stage can therefore be attributed to that. The CW matrix displayed a curve between that of the casein matrices and the W matrix. This outcome was expected as CW contained both whey proteins and caseins. By the end of the intestinal phase, the differences observed at the gastric level were strongly attenuated as all proteins were completely hydrolyzed. These results are again in accordance with those obtained in the studies by Egger et al.

The peptide patterns of the matrices illustrated in Figure 4A show that protein hydrolysis of α-S1-casein starts at the gastric phase and that a lot of large peptides are generated starting from the 16^th^ amino acid of the protein sequence. At the end of the intestinal phase, a variety of smaller-sized peptides are generated, showing that α-S1 casein exhibits a multitude of small resistant zones. When looking at β-lactoglobulin (Figure 4B), the peptide pattern obtained during digestion is different. Indeed, the protein is very lightly hydrolyzed by the end of the gastric phase. However, by the end of the intestinal phase, the protein seems to be more hydrolyzed than α-s1-casein as it exhibits fewer resistant zones, and β-lactoglobulin seems to majorly exhibit peptides made up of 16 amino acids. All matrices seem to follow the same pattern, and the differences observed in amino acid occurrences is due to the distribution of each protein in every matrix. In a study where human volunteers ingested whey proteins and caseins, whey generated a majority of 9–15 amino acid residue peptides while caseins generated mainly 6–9 amino acid residue peptides [22]. This is similar to what is observed in our study as α-s1-casein generates smaller amino acid residue peptides than β-lactoglobulin. An important limitation to note in this study is that only peptides that were between 500 to 2500 Da were identified in this context. Indeed, one of the limits of LC-MS/MS when studying digestive peptidomes is that small-sized and large-sized peptides often go undetected [23]. Therefore, some existing differences in peptide patterns regarding very small and large peptides might not be perceived in this study.

### 4.3. Peptide Identification During Digestion of the Matrices

Using a more resolutive and sensitive analytical method (HPLC-MS/MS and bioinformatics), more differences are observed between the matrices in terms of peptides detected. Indeed, at the end of the gastric phase (Figure 5A), three distinct clusters are distinguished: one consisting of the W matrix replicates, one formed of the C3 replicates, and a third one regrouping the rest of the matrices. As previously explained above, the W matrix is different from the other matrices as it is constituted mainly of native whey proteins. Thus, the generated peptides are vastly different from those obtained after hydrolysis of casein matrices. However, two unique peptides generated from beta-casein were identified. This shows that trace amounts of caseins are still present in the W matrix, even after the filtration process. The CW matrix appears in the same cluster as the casein matrices as it contains a higher proportion of caseins than whey proteins and thus generated more casein-based peptides. Moreover, a result that is observed here and that was not highlighted before is that the C3 matrix stands out. This means that the peptides generated at the gastric phase are different due to the enzymatic process that was applied in order to reticulate the caseins present in the C3 matrix. Reticulated casein micelles have a different structure than native casein micelles as they are smaller in size and have a smoother surface [24]. Moreover, it could be hypothesized that the cross-linkage induced between the caseins may either prevent the enzymes from accessing the same sites as the ones of the native casein micelles due to steric hindrance or that the enzymatic process restricted the cleavage reactional mechanism. At the end of the intestinal phase, besides the W and C3 matrices which were already distinguished from the other matrices, the C2 low Ca^2+^ replicates also form a cluster of their own. This matrix has the lowest concentration in calcium, and as previously mentioned, caseins interact together to form micelles. These interactions are allowed through the action of calcium ions that form linkages between the casein molecules either as colloidal calcium phosphate by binding directly to caseins [2,25,26]. It has already been established that removing colloidal calcium phosphate by processes such as acidification or a calcium chelator could disintegrate the micelle into smaller particles [27]. Disintegration of the casein micelle could thus play a role in generating peptides different from those of the other casein matrices. Moreover, the C2 low Ca^2+^ matrix generated the greatest number of highest mean normalized abundance peptide sequences from all four casein types, whereas C2 and C3 only generated two and three highest mean normalized abundance peptides respectively. The β-casein was the biggest precursor of peptides, generating a total of 23 highest mean normalized abundance peptides. These results are in accordance with those performed in the human trial by Boutrou et al., where β-casein was the largest precursor of peptides [28].

When all the chemical data were compiled with the amino acid occurrences for each protein matrix at the end of each phase (Figure 6 and Figure 7), the same global tendencies were observed, with some slight differences. At the end of the gastric phase (Figure 6A), the W and C3 matrices were different from the other matrices, a trend which was already noted in the mass spectrometry data in Figure 5. This again can be explained by the fact that the W matrix contained a different category of proteins (native whey proteins) and that the C3 matrix was modified enzymatically. However, the CCSWA analysis further showed a distinction of the CW matrix which was not observed in the mass spectrometry data (Figure 5). At the end of the intestinal phase (Figure 7A), the W matrix was clearly distinct from the other matrices on the CC1 axis. However, the replicates of all the other matrices were very dispersed on the CC2 axis. As the chemical data were used to construct the CC2 axis, it can be said that differences at the levels of the molecular weights generated, peptide concentrations, calcium concentrations, and total free amino acid concentrations were noted between all the matrices. It can thus be hypothesized that the industrial process has an effect not only on protein distribution but also on the protein conformation, which leads to differences during in vitro digestion. Moreover, the additional differences that arose between the matrices with the CCSWA analysis highlight the utility of this method, which can serve as a complementary analysis that allows for the comparison of data with different number of variables.

## 5. Conclusions

This study first demonstrated that, although the INFOGEST protocol is the method of choice when simulating static in vitro digestion, some adjustments can still be necessary when trying to resolve certain research questions, in this case, the quantity of pancreatin needed to be optimized in order to better adapt it to our simple milk protein matrices. By digesting our milk matrices with pancreatin harboring a trypsin activity of 27.3 U.mL^−1^ instead of 100 U.mL^−1^, similar results were observed in terms of peptide molecular weight and electrophoretic profiles. These profiles also showed the importance of pancreatin autolysis when large amounts of pancreatin were used. This study is also further proof that whey proteins are resistant to gastric digestion but are then very hydrolyzed by the end of the intestinal phase. The industrial process affected not only the protein distribution of the matrices but also most likely the protein structure. Indeed, this study showed that. by reticulating the caseins or by reducing their calcium concentrations, statistical qualitative and quantitative differences arose in terms of peptides generated in the gastric and intestinal compartments, which could lead to differences in term of amino acid bioavailability and peptide bioactivities. These results shall be confirmed in vivo or by using a dynamic in vitro digestion method to confirm the relevance of using the quick and less costly static method to characterize the effect of industrial processes on milk matrices’ digestion.

## Figures and Tables

**Table 1:** Characteristics of the milk matrices.

	Native Micellar Casein (%)	Whey Proteins (%)	Calcium (%)	Total Nitrogen Content (%)
C1	92	8	2.6	83
C2	92	8	2.1	82.3
C2 low Ca^2+^	92	8	1.6	85.1
C3	92	8	2.6	80.1
CW	80	20	2.2	81
W	0	100	0.3	81.5

C: casein; W: whey. Matrices were color coded: C1; purple, C2; dark orange, C2 low Ca2+; light orange, C3; light blue, CW; dark blue, and W; green. This color code was adopted for the entire study. The matrices C1, C2, and C3 were all built up native micellar caseins. C1 and C2 differed only in their calcium concentrations. C3 had the same calcium concentration as C1 but was obtained by an enzymatic process which reticulated the caseins.

**Table 2:** Chemical data of the different matrices at the end of the gastric phase and at the end of the intestinal phase.

Milk Matrices	Phase	MW > 10 kDa	4 < MW < 10 kDa	2 < MW < 4 kDa	0.7 < MW < 2 kDa	0.3 < MW < 0.7 kDa	MW < 3 kDa	Peptide Concentration (mg/mL^−1^)	Calcium Concentration (mg/mL^−1^)	Total Free AA Concentration (mg/100g of Product)
C1	Gastric	4.9 *±* 0.7	43.9 *±* 0.7	27.9 *±* 0.8	18.0 *±* 0.0	4.7 *±* 0.4	0.6 *±* 0.2	0.24	0.7	<LD
C2		5.7 *±* 0.5	45.4 *±* 0.7	26.3 *±* 1.1	18.0 *±* 0.3	4.3 *±* 0.6	0.7 *±* 0.2	0.33 *±* 0.04	0.6	<LD
C2 low Ca^2+^		7.6 *±* 1.3	43.6 *±* 4.2	25.6 *±* 0.9	18.5 *±* 0.9	4.3 *±* 1.0	0.6 *±* 0.5	0.16	0.4	<LD
C3		5.8 *±* 4.1	41.9 *±* 1.3	26.4 *±* 1.1	20.9 *±* 2.7	4.5 *±* 0.4	0.5 *±* 0.1	0.26	0.8	<LD
CW		8.8 *±* 0.4	43.9 *±* 0.3	25.9 *±* 0.4	16.0 *±* 0.5	5.1 *±* 0.4	0.5 *±* 0.2	0.28 *±* 0.01	0.5	<LD
W		17.9 *±* 2.2	23.5 *±* 5.1	22.1 *±* 0.6	27.3 *±* 3.8	8.5 *±* 3.6	1.2 *±* 0.0	0.16	0.1	<LD
C1	Intestinal	0.6 *±* 0.1	4.0 *±* 0.5	13.4 *±* 1.5	47.5 *±* 1.1	20.0 *±* 0.8	14.9 *±* 0.6	0.33	0.5	171.2 *±* 32.7
C2		0.7 *±* 0.1	4.3 *±* 0.3	15.0 *±* 0.3	46.7 *±* 1.0	17.8 *±* 0.6	15.9 *±* 0.6	0.41 *±* 0.02	0.4	189.0 *±* 55.3
C2 low Ca^2+^		0.7 *±* 0.1	4.5 *±* 0.3	15.6 *±* 0.6	45.4 *±* 0.7	17.2 *±* 2.4	17.2 *±* 1.5	0.36	0.3	233.3 *±* 4.5
C3		0.9 *±* 0.2	6.0 *±* 1.4	14.9 *±* 0.9	44.6 *±* 1.2	18.0 *±* 0.9	15.7 *±* 0.8	0.32	0.5	148.0 *±* 27.5
CW		0.7 *±* 0.3	4.2 *±* 0.2	14.6 *±* 0.3	44.3 *±* 2.1	21.7 *±* 1.5	15.0 *±* 1.5	0.34 *±* 0.01	0.3	253.3 *±* 15.0
W		1.1 *±* 0.1	3.3 *±* 0.2	9.7 *±* 0.2	40.6 *±* 0.1	26.6 *±* 0.7	18.8 *±* 0.0	0.36	0.1	277.3 *±* 4.6

Results are presented as mean ± SD when samples were analyzed as triplicates and as a single value when only one replicate was used for analysis. MW: molecular weight; LD: Limit of detection, AA: amino acid.

## Supplementary Materials

The following are available online at https://www.mdpi.com/2304-8158/9/11/1580/s1, Figure S1: Molecular weight profiles of gastric and intestinal digests., Table S1: Exhaustive list of identified peptides.

## References

[1] Fox P.F. (2001). Milk proteins as food ingredients. Int. J. Dairy Technol..

[2] Dalgleish D., Corredig M. (2012). The Structure of the Casein Micelle of Milk and Its Changes During Processing. Annu. Rev. Food Sci. Technol..

[3] Lacroix M., Bos C., Léonil J., Airinei G., Luengo C., Daré S., Benamouzig R., Fouillet H., Fauquant J., Tomé D. (2006). Compared with casein or total milk protein, digestion of milk soluble proteins is too rapid to sustain the anabolic postprandial amino acid requirement. Am. J. Clin. Nutr..

[4] Mahé S., Roos N., Benamouzig R., Davin L., Luengo C., Gagnon L., Gaussergès N., Rautureau J., Tomé D. (1996). Gastrojejunal kinetics and the digestion of [15N]beta-lactoglobulin and casein in humans: The influence of the nature and quantity of the protein. Am. J. Clin. Nutr..

[5] Boirie Y., Dangin M., Gachon P., Vasson M.-P., Maubois J.-L., Beaufrère B. (1997). Slow and fast dietary proteins differently modulate postprandial protein accretion. Proc. Natl. Acad. Sci. USA.

[6] Van Lieshout G.A.A., Lambers T.T., Bragt M.C.E., Hettinga K.A. (2019). How processing may affect milk protein digestion and overall physiological outcomes: A systematic review. Crit. Rev. Food Sci. Nutr..

[7] Coskun A.E.I., Sağlam D., Venema P., Van Der Linden E., Scholten E. (2015). Preparation, structure and stability of sodium caseinate and gelatin micro-particles. Food Hydrocoll..

[8] Dalgleish D., Spagnuolo P.A., Goff H.D. (2004). A possible structure of the casein micelle based on high-resolution field-emission scanning electron microscopy. Int. Dairy J..

[9] Wang X., Ye A., Lin Q., Han J., Singh H. (2018). Gastric digestion of milk protein ingredients: Study using an in vitro dynamic model. J. Dairy Sci..

[10] Minekus M., Alminger M., Alvito P., Ballance S., Bohn T., Bourlieu C., Carrière F., Boutrou R., Corredig M., Dupont D. (2014). A standardised staticin vitrodigestion method suitable for food—An international consensus. Food Funct..

[11] Egger L., Schlegel P., Baumann C., Stoffers H., Guggisberg D., Brügger C., Dürr D., Stoll P., Vergères G., Portmann R. (2017). Physiological comparability of the harmonized INFOGEST in vitro digestion method to in vivo pig digestion. Food Res. Int..

[12] Bohn T., Carriere F., Day L., Deglaire A., Egger L., Freitas D., Golding M., Le Feunteun S., Macierzanka A., Ménard O. (2017). Correlation between in vitro and in vivo data on food digestion. What can we predict with static in vitro digestion models?. Crit. Rev. Food Sci. Nutr..

[13] Egger L., Ménard O., Baumann C., Duerr D., Schlegel P., Stoll P., Vergères G., Dupont D., Portmann R. (2019). Digestion of milk proteins: Comparing static and dynamic in vitro digestion systems with in vivo data. Food Res. Int..

[14] Bidlingmeyer B.A., Cohen S.A., Tarvin T.L. (1984). Rapid analysis of amino acids using pre-column derivatization. J. Chromatogr. B Biomed. Sci. Appl..

[15] Laemmli U.K. (1970). Cleavage of Structural Proteins during the Assembly of the Head of Bacteriophage T4. Nat. Cell Biol..

[16] Evincent D., Elkins A., Condina M.R., Ezernieks V., Rochfort S. (2016). Quantitation and Identification of Intact Major Milk Proteins for High-Throughput LC-ESI-Q-TOF MS Analyses. PLoS ONE.

[17] Cordella C.B., Bertrand D. (2014). SAISIR: A new general chemometric toolbox. Trends Anal. Chem..

[18] Mandalari G., Adel-Patient K., Barkholt V., Baro C., Bennett L., Bublin M., Gaier S., Graser G., Ladics G., Mierzejewska D. (2009). In vitro digestibility of β-casein and β-lactoglobulin under simulated human gastric and duodenal conditions: A multi-laboratory evaluation. Regul. Toxicol. Pharmacol..

[19] Zhao Y., Lin Y.-H. (2010). Whole-Cell Protein Identification Using the Concept of Unique Peptides. Genom. Proteom. Bioinform..

[20] Ménard O., Famelart M.-H., Deglaire A., Le Gouar Y., Guérin S., Malbert C.-H., Dupont D. (2018). Gastric Emptying and Dynamic In Vitro Digestion of Drinkable Yogurts: Effect of Viscosity and Composition. Nutrients.

[21] Qiao Y., Gumpertz M., Van Kempen T. (2005). Stability of a Pancreatic Enzyme Cocktail during in Vitro Protein Digestibility Assays. J. Food Biochem..

[22] Stewart R.J.C., Morton H., Coad J., Pedley K.C. (2018). In vitro digestion for assessing micronutrient bioavailability: The importance of digestion duration. Int. J. Food Sci. Nutr..

[23] De Cicco M., Mamone G., Di Stasio L., Ferranti P., Addeo F., Picariello G. (2019). Hidden “Digestome”: Current Analytical Approaches Provide Incomplete Peptide Inventories of Food Digests. J. Agric. Food Chem..

[24] Broyard C., Gaucheron F. (2015). Modifications of structures and functions of caseins: A scientific and technological challenge. Dairy Sci. Technol..

[25] Holt C. (1992). Structure and Stability of Bovine Casein Micelles. Adv. Protein Chem..

[26] Tsioulpas A., Lewis M.J., Grandison A.S. (2007). Effect of Minerals on Casein Micelle Stability of Cows’ Milk. J. Dairy Res..

[27] Fox P., Brodkorb A. (2008). The casein micelle: Historical aspects, current concepts and significance. Int. Dairy J..

[28] Boutrou R., Gaudichon C., Dupont D., Jardin J., Airinei G., Marsset-Baglieri A., Benamouzig R., Tome D., Leonil J. (2013). Sequential release of milk protein-derived bioactive peptides in the jejunum in healthy humans. Am. J. Clin. Nutr..

