# Supplementary material for: In Vitro Assessment of the Impact of Industrial Processes on the Gastrointestinal Digestion of Milk Protein Matrices Using the INFOGEST Protocol
Source: Foods. 2020 Oct 30;9(11):1580. doi: 10.3390/foods9111580 (PMC7693224; doi:10.3390/foods9111580)
Supplement: Supplementary file 1 [file foods-09-01580-s001.zip › Figure S1.docx]

**Supplemental Figure**

**
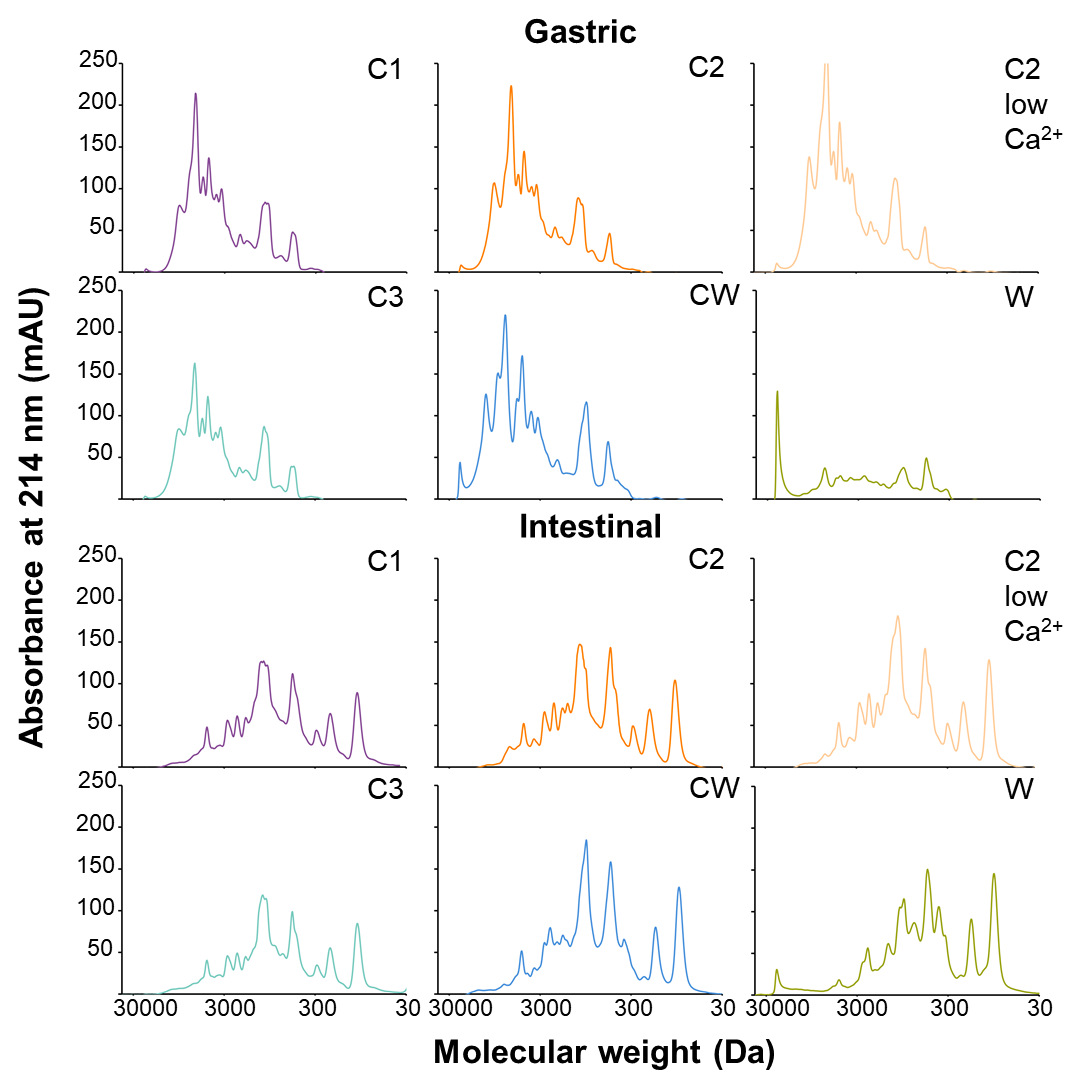
**

**Figure S1. Molecular weight profiles of gastric and intestinal digests.** Peptide profiles were obtained by size exclusion chromatography-fast protein liquid chromatography (SEC-FPLC): 2h-gastric digests (top) and 2h-intestinal digests (bottom).
