# Supplementary material for: In Vitro Assessment of the Impact of Industrial Processes on the Gastrointestinal Digestion of Milk Protein Matrices Using the INFOGEST Protocol
Source: Foods. 2020 Oct 30;9(11):1580. doi: 10.3390/foods9111580 (PMC7693224; doi:10.3390/foods9111580)
Supplement: Supplementary file 1 [file foods-09-01580-s001.zip › Table S1.docx]

**Table S1**: **Exhaustive list of identified peptides.**

Exhaustive list of identified peptides in intestinal digests displaying a mass signal intensity > to 1^E^5 and an ANOVA p-value < to 0.005. Amino acid sequences are classified according to i) the dairy matrices where the identified peptides are present in highest mean quantity, ii) the milk proteins, iii) the ANOVA p-value and iv) the maximum fold change.

Ser + 79.97 (Phosphorylation); N + 21.98 (sodium adduct); E + 21.98 (sodium adduct); G + 14.02 Gly to Ala; Q + 17.03 (replacement of a proton by ammonium ion)
